# Mechanism and therapeutic potential of liver injury induced by cholesterol-associated proteins

**DOI:** 10.3389/fphar.2025.1572592

**Published:** 2025-03-27

**Authors:** Yourong Zhou, Yashi Cao, Yiming Yin, Zhifei Xu, Xiaochun Yang, Bo Yang, Peihua Luo, Hao Yan, Qiaojun He

**Affiliations:** ^1^ Center for Drug Safety Evaluation and Research of Zhejiang University, College of Pharmaceutical Sciences, Zhejiang University, Hangzhou, Zhejiang, China; ^2^ Institute of Pharmacology and Toxicology, College of Pharmaceutical Sciences, Zhejiang University, Hangzhou, Zhejiang, China; ^3^ School of Medicine, Hangzhou City University, Hangzhou, Zhejiang, China; ^4^ Innovation Institute for Artificial Intelligence in Medicine of Zhejiang University, Hangzhou, Zhejiang, China

**Keywords:** cholesterol metabolism, liver injury, cholesterol-related proteins, regulatory factors, therapeutic targets

## Abstract

Cholesterol, the most abundant sterol molecule in mammalian organisms, serves not only as a fundamental structural component of cell membranes but also as a critical regulator of cellular signaling and function. Cholesterol-associated proteins can mediate liver injury either directly by influencing cholesterol levels or through non-cholesterol pathways. These non-cholesterol pathways, which operate independently of cholesterol’s traditional metabolic functions, are regulated by specific transcription factors, proteins and receptors. Dysregulation of cholesterol-associated can disrupt cellular homeostasis, leading to liver injury, metabolic disorders, and even tumorigenesis. In this article, we explore the mechanisms by which cholesterol-associated proteins contribute to liver injury via both classical cholesterol pathways and non-cholesterol pathways, and discuss their potential as therapeutic targets for liver-related diseases.

## 1 Introduction

Cholesterol serves as a critical structural component of mammalian cell membranes, cellular architecture, signal transduction cascades, endocytic processes, receptor modulation, and cytoskeletal dynamics. Thus, intracellular cholesterol concentrations are tightly regulated by complex processes including cholesterol biosynthesis, uptake, efflux, trafficking, and esterification (Nguyen et al., 2024). The liver is pivotal in the overall cholesterol metabolism process. Beyond its role as a primary site for cholesterol biosynthesis, the liver actively contributes to bile acid synthesis and cholesterol trafficking. Key regulatory proteins governing cholesterol metabolism are instrumental in maintaining cholesterol homeostasis and the health status of the liver, suggesting a potential association between these regulatory proteins and the development together with the progression of liver pathologies. Therefore, this study aims to elucidate and evaluate the role of cholesterol metabolism-related proteins in liver injuries, discussing the molecular mechanisms of cholesterol-related proteins involved in metabolism and their potential as therapeutic targets for liver injuries.

## 2 Overview of cholesterol metabolism

### 2.1 Cholesterol metabolic processes

**FIGURE 1:**
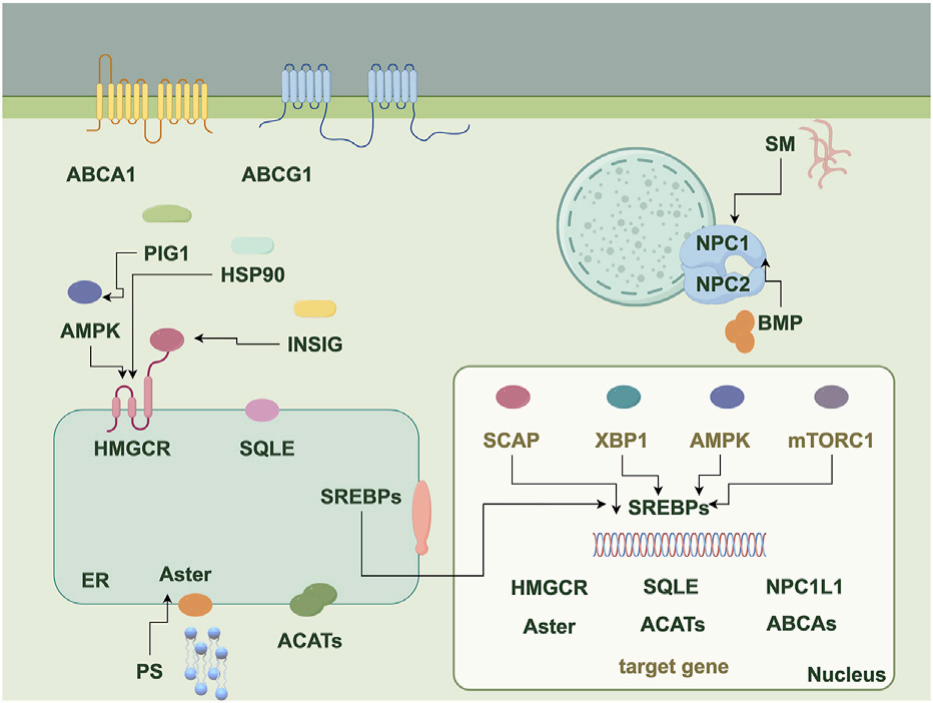
The diagram illustrates proteins, molecules, and pathways related to cholesterol metabolism. It serves as a flowchart depicting the various proteins, molecules, and pathways involved in the process, highlighting the interactions among these components in cholesterol metabolism, including endogenous synthesis, exogenous uptake, transport, esterification, and efflux. ABCA1, ATP-binding cassette transporter A1; ABCG1, ATP-binding cassette transporter G1; SM, Sphingomyelin; NPC1/2, Niemann-Pick disease type C1/2; BMP, bis (monoacylglycerol) phosphate; PIG1, Protein interaction gene 1; AMPK, AMP-activated protein kinase; HSP90, Heat shock protein 90; INSIG, Insulin-induced gene; SCAP, SREBP cleavage activating protein; XBP1, X-box binding protein 1; mTORC1, Mechanistic target of rapamycin complex 1; SREBPs, Sterol regulatory element binding proteins; HMGCR, 3-hydroxy-3-methyl glutaryl coenzyme A reductase; SQLE, Squalene epoxidase; NPC1L1, Niemann-Pick C1-like 1; ACATs, Acetyl coenzyme A acetyltransferases; PS, Phosphatidylserine.

The homeostasis of cholesterol metabolism is important for the physiological functions of the organism. Cholesterol metabolism consists of five processes: endogenous synthesis, exogenous uptake, transport, esterification, and efflux. Endogenous cholesterol biosynthesis occurs predominantly in the liver, where the substrate acetyl-CoA is converted via the mevalonic acid (MVA) pathway to generate structural precursors of sterol substances. Within this process, 3-hydroxy-3-methyl glutaryl coenzyme A reductase (HMGCR) serves as the rate-limiting enzyme of the MVA pathway and is also the target of cholesterol-lowering drugs such as statins (Yu and Liao, 2022). Under the catalysis of farnesyl diphosphate synthase and squalene synthase, the structural precursors of sterol substances are transformed into squalene (SQ), which represents the biochemical precursor for all terpenes and steroids (Gohil et al., 2019). Subsequently, SQ is oxidized by squalene epoxidase (SQLE) to form 2,3-oxidosqualene (Zhang et al., 2019), representing another key step in cholesterol biosynthesis. Additionally, dietary uptake is another pathway for mammals to acquire cholesterol, where these cholesterol molecules are taken up by epithelial cells through vesicular endocytosis mediated by Niemann-Pick C1-like 1 (NPC1L1) (Ge et al., 2011; Zeng et al., 2022; Altmann et al., 2004). NPC1L1 transports free extracellular cholesterol to the cell membrane, then recruits Aster to the cell membrane and initiates cholesterol transport from the cell membrane to the endoplasmic reticulum (ER) when the cholesterol level on the cell membrane is elevated. Oxysterol-binding protein (OSBP) and related protein homologue ORPs constitute a family of lipid-transfer proteins that operate at membrane contact sites (Mesmin and Antonny, 2016). The transport of cholesterol involves multiple steps. Within cells, OSBP is responsible for transferring cholesterol from the ER to the trans-Golgi network. In the body, cholesterol transport is primarily facilitated by chylomicrons, which carry cholesterol from intestinal epithelial cells through lymphatic vessels into the bloodstream. Cholesterol in the blood is then taken up by cells via the low-density lipoprotein receptor (LDLR) through a process known as endocytosis. Upon its entry into the cell, LDL dissociates from LDLR in the endosome and later enters the lysosome. Free cholesterol is released from lysosomes and to prevent free cholesterol accumulation, acetyl coenzyme A acetyltransferase 1 (ACAT1) transfers long-chain fatty acids to cholesterol thereby forming cholesteryl esters by esterification and binding into cytoplasmic lipid droplets (Qian et al., 2020b). In most cell types, excess cholesterol is initially transported out of the cell by ATP-binding cassette transporter 1 (ABCA1), which facilitates the efflux of lipids including phospholipids and cholesterol. Subsequently, these lipids bind to extracellular lipid-binding proteins, leading to the formation of nascent high-density lipoprotein (HDL) (Luo et al., 2020). Furthermore, the transcription factor sterol regulatory element binding proteins (SREBPs), which are extensively regulated at multiple levels, play a key role in regulating the overall homeostasis of cholesterol (Luo et al., 2020). The complete cholesterol metabolism process and associated regulatory factors are shown in Figure 1.

**FIGURE 2:**
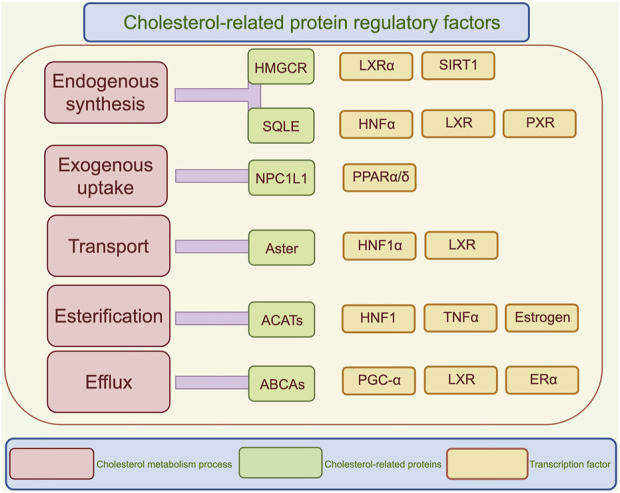
Regulatory mechanisms of cholesterol metabolism. This diagram illustrates the regulatory factors related to cholesterol, categorized into five processes: endogenous synthesis, exogenous uptake, transport, esterification, and efflux. Each process lists key enzymes and transcription factors, highlighting the complexity of cholesterol metabolism and its regulatory mechanisms. SREBPs, Sterol regulatory element binding proteins; HMGCR, 3-hydroxy-3-methyl glutaryl coenzyme A reductase; SQLE, Squalene epoxidase; NPC1L1, Niemann-Pick C1-like 1; ACATs, Acetyl coenzyme A acetyltransferases; LXRα, liver X receptor-α; SIRT1, Silent information regulator 1; HNFα, Hepatic nuclear factor α; PXR, Pregnane X receptor; PPARα/δ, peroxisome proliferators activated receptor α/δ; TNFα, Tumor necrosis factor; PGC-1α, Peroxisome proliferators-activated receptor γ coactivator α; ERα, Estrogen receptor α.

Hepatic cholesterol homeostasis is coordinately regulated by nutrition, the circadian clock, and the endocrine system. High intake of saturated fatty acids in the diet activates the Toll-like receptor 4 signaling pathway on hepatocyte membranes, modulating the expression of SREBP1, which subsequently upregulates the expression of LDLR and HMGCR (Oishi et al., 2017). Conversely, phytosterols exert a counter-regulatory effect through the gut microbiota (Lv et al., 2023). The hypothalamic-pituitary axis regulates cholesterol metabolism via the circadian secretion of corticosterone, and clinical studies have revealed that shift workers face an increased risk of hepatic cholesterol accumulation due to circadian disruption (Rao and Xue, 2024). Glucocorticoids mediate chromatin remodeling, enhancing the promoter activity of ABCA1 and promoting cholesterol efflux (Fadel et al., 2023). Notably, estrogen exerts sex-specific modulation of these pathways through estrogen receptor alpha, providing a molecular explanation for the surge in metabolic dysfunction-associated steatotic liver disease (MASLD) incidence among postmenopausal women (Hart-Unger et al., 2017).

Cholesterol metabolism is closely intertwined with glucose and lipid metabolism. In glucose metabolism, insulin regulates the SREBP pathway through mTOR-dependent transcriptional and post-transcriptional mechanisms (DeBose-Boyd and Ye, 2018). Conversely, ER stress induced by high cholesterol levels can trigger a vicious cycle of insulin resistance via the IRE1-XBP1 pathway (Flamment et al., 2012). In lipid metabolism, liver X receptors (LXRs), upon sensing elevated oxysterol levels, not only activate ABCA1 transporters to promote cholesterol efflux but also drive fatty acid synthesis by inducing the expression of fatty acid synthase (FASN) and stearoyl-CoA desaturase-1 (SCD1) (Wang and Tontonoz, 2018). Figure 2 illustrates the effects of various regulatory factors on the different metabolic processes of cholesterol.

### 2.2 Effects of cholesterol in liver injury

Metabolic dysfunction-associated fatty liver disease (MAFLD) is a spectrum of liver alterations that begins with the accumulation of fat in the liver (hepatocellular steatosis occupies >5% of the range of liver tissue specimens obtained) in the absence of alcohol, viral infections, or drugs that promote steatosis (abnormal retention of fat) and can further evolve into metabolic dysfunction-associated steatohepatitis (MASH), liver fibrosis, and eventually cirrhosis and hepatocellular carcinoma (HCC) (Chalasani et al., 2018). Steatosis is the first event in the development of MAFLD. During the progression from MAFLD to MASH, several lipid substances are stored in hepatocytes, including triacylglycerol, diacylglycerol, free fatty acids (FFAs), ceramides, and cholesterol. Thus, imbalances in lipid metabolism are the most direct contributors to the formation of certain liver injuries (Younossi et al., 2021). Recently, with a clearer definition of the regulatory function of the mechanistic pathways, hepatic free cholesterol and its metabolite-mediated hepatocytotoxicity have been recognized as the basis of subsequent necrotic inflammation and fibrosis in MAFLD (Kakiyama et al., 2023).

Existing studies have found that pathogen-associated and hazard-associated molecular patterns, such as lipopolysaccharide and cholesterol crystals (Kelley et al., 2019) may penetrate disturbed tight junctions and promote the activation of NOD-like receptor family pyrin domain containing 3 (NLRP3) inflammatory vesicles in hepatocytes (Ioannou et al., 2013). In contrast, the use of benzyl isothiocyanate reduced lipopolysaccharide and cholesterol crystal-induced activation of NLRP3 inflammatory vesicles in Kupper cells and prevented the development of MASH (Chen et al., 2020). Additionally, cholesterol metabolism emerges as a critical determinant in the progression from MASH to liver fibrosis, orchestrating inflammatory responses in the liver through the activation of the NLRP3 inflammasome. Apart from Kupffer cells, cholesterol accumulation in hepatic stellate cells has been identified as an important player in the development of MASH and liver fibrosis (Tomita et al., 2014a; Teratani et al., 2012). Moreover, recent evidence suggests that cholesterol-rich diets can also promote the development of MASH by activating gastrin processing (Koh et al., 2021). Following the administration of a high-fat, high-cholesterol diet, transgenic mice with intestinal-specific hyperactivation of SREBP2 develop hepatic steatosis and show a higher susceptibility to fibrosis in response to hepatic injury, and inflammation (Malhotra et al., 2017).

HCC, the end-stage manifestation of chronic liver disease primarily driven by viral hepatitis or MASH, is the fourth leading cause of cancer-related deaths worldwide due to late diagnosis and poor treatment outcomes (Llovet et al., 2021; Choi et al., 2022; Marchi et al., 2020). In this context, besides being identified at the onset of MASH (Ribas et al., 2021; Ioannou, 2016), it has also been found that elevation of hepatic cholesterol in the progression of MASH to HCC plays a key role in tumor initiation (Liu et al., 2018; Bakiri et al., 2017; Liang et al., 2018). Compared to mice fed a high-fat diet, the presence of cholesterol in the diet enhances MASH-driven HCC, suggesting that it is cholesterol itself rather than steatosis that promotes the progression from MASH to HCC (Conde De La Rosa et al., 2021). Consistently, mitochondrial cholesterol loading impairs the outer mitochondrial membrane, affecting the oligomerization of anti-apoptotic proteins and subsequently promoting tumor growth in HCC (Montero et al., 2008).

## 3 Cholesterol-associated proteins and impact on liver injury

**FIGURE 3:**
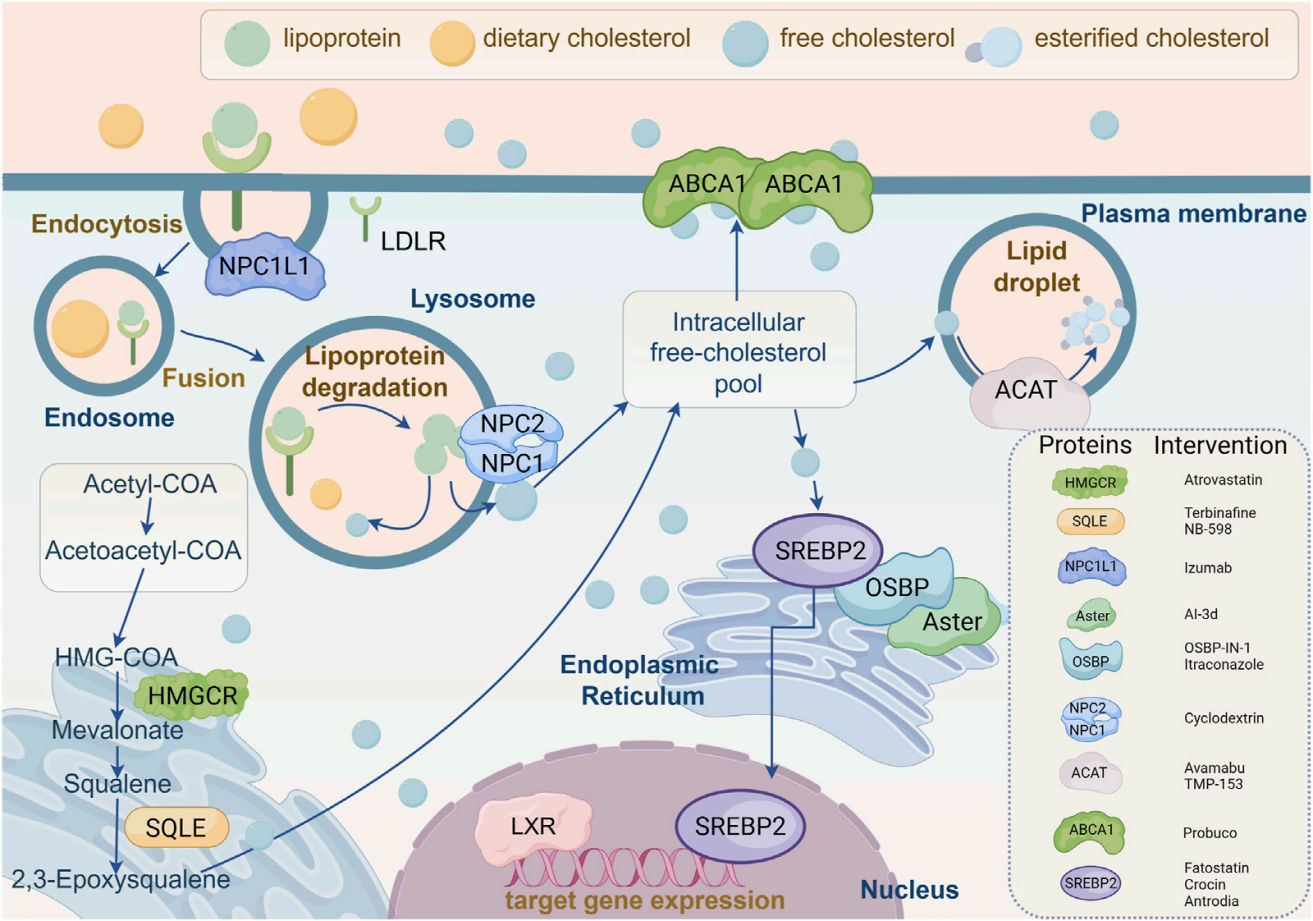
Molecular mechanisms of cholesterol-related target genes and proteins regulating cholesterol biosynthesis. This figure provides a comprehensive overview of cellular cholesterol homeostasis, illustrating the complexity of cholesterol uptake, intracellular transport, biosynthesis, and efflux. The diagram depicts the internalization of lipoproteins and dietary cholesterol via low-density lipoprotein receptor (LDLR). Following internalization, lipoproteins are degraded in lysosomes to release free cholesterol, which is then distributed to the intracellular pool of free cholesterol by Niemann-Pick C1 (NPC1) and NPC2 proteins. Free cholesterol can be esterified by acetyl coenzyme A acetyltransferase (ACAT) for storage in lipid droplets, or effluxed from the cell via ATP-binding cassette transporter A1 (ABCA1). Cholesterol biosynthesis is elucidated in the lower left quadrant, occurring in the endoplasmic reticulum and involving key enzymes such as 3-hydroxy-3-methyl glutaryl coenzyme A reductase (HMGCR) and squalene epoxidase (SQLE), through intermediates such as mevalonic acid and squalene. The core mechanisms regulating cholesterol synthesis and transport are emphasized in the diagram, involving sterol regulatory element-binding protein 2 (SREBP2), oxysterol-binding protein (OSBP), and Aster. Additionally, specific proteins involved in cholesterol metabolism and their corresponding inhibitors are listed in the figure, such as atorvastatin targeting HMGCR, terbinafine and NB-598 targeting SQLE, and various other inhibitors targeting NPC1L1, ACAT, ABCA1, and SREBP2. At the bottom, the role of liver X receptor (LXR) in regulating the expression of cholesterol turnover-related target genes is illustrated. In conclusion, this schematic summarizes the dynamic processes of intracellular cholesterol management, highlighting potential pharmacological targets for regulating cholesterol levels and addressing dyslipidemia. NPC1L1, Niemann-Pick C1-like 1; LDLR, Low-density lipoprotein receptor; NPC1/2, Niemann-Pick C1/2; HMGCR, 3-hydroxy-3-methyl glutaryl coenzyme A reductase; SQLE, Squalene epoxidase; ABCA1, ATP-binding cassette transporter A1; SREBP2, Sterol regulatory element-binding protein 2; OSBP, Oxysterol-binding protein; LXR, Liver X receptor; ACAT, Acetyl coenzyme A acetyltransferase.

Dysregulated expression of cholesterol-related genes and target proteins disrupts classical cholesterol metabolism, promoting hepatocyte death, carcinogenesis, or proliferation by altering cholesterol levels. Current studies have reported that these target genes and proteins can also directly regulate the pathological progression of liver injury independently of cholesterol. Therefore, elucidating the regulatory mechanisms, roles in liver disease, and therapeutic targeting potential of these genes and proteins may lay the groundwork for innovative treatment strategies. The position of each key protein in cholesterol metabolism is depicted in Figure 3.

### 3.1 HMGCR

#### 3.1.1 Structure of the HMGCR

HMGCR is a glycoprotein containing 887 amino acid residues, primarily localized in the ER of the liver and intestine. It consists of a transmembrane structural domain and a catalytic structural domain that extends into the cytoplasm (Li et al., 2020). Its hydrophobic N-terminus contains eight transmembrane domains, including a sterol-sensing domain responsible for sensing cholesterol levels. When cytoplasmic cholesterol levels are elevated, insulin-induced gene-encoded proteins bind to the sterol-sensing domain on HMGCR, leading to its degradation and further inhibition of cholesterol synthesis. The hydrophilic C-terminus directly impacts the activity of HMGCR (Jo and Debose-Boyd, 2010).

#### 3.1.2 Regulatory factors of HMGCR level

At the molecular level, transcription factors such as SREBP2 and liver X receptor-α (LXR-α) regulate the transcriptional level of HMGCR by binding to its promoter region. External stimuli such as drugs can also affect HMGCR expression. For instance, dexamethasone can enhance intracellular and extracellular total cholesterol levels in bone marrow mesenchymal stem cell-derived hepatocyte-like cells and human liver cancer cells HepG2, leading to a reduction in the expression of silencing information regulator 1, thereby facilitating *HMGCR* histone acetylation and expression (Li et al., 2022a). Electroacupuncture attenuates HMGCR protein expression by suppressing its deubiquitination (Jin et al., 2023). In addition, several studies have revealed that HMGCR can be phosphorylated and inactivated by cytoplasmic protein kinases such as adenosine 5′-monophosphate-activated protein kinase (AMPK), and inactivated HMGCR can be catalytically dephosphorylated by phosphodiesterase in the cytoplasm to restore enzymatic activity (Jo and Debose-Boyd, 2010). Retinoic acid induced gene I (RIG-I) is a sensor that recognizes RNA viruses in innate immune cells and is mainly expressed by hepatic parenchymal cells in the liver. Constitutively monomethylated modification of RIG-I occurs at the K18 and K146 sites, and constitutively methylated RIG-I bound to AMPK inhibits the phosphorylation of HMGCR (Li et al., 2022b). miRNAs are the most abundant class of small, naturally occurring non-coding RNAs in animals, consisting of 19–25 nucleotides, and are important regulators that affect gene expression (Liu et al., 2023). miRNAs such as miR-192 have also been found to affect the translation and degradation process by targeting HMGCR mRNA (Lin et al., 2017).

#### 3.1.3 HMGCR as a therapeutic target in liver injury

In patients with MAFLD and MASLD, the mRNA expression level of HMGCR increased 2–3 times, and the protein level of HMGCR increased 3–4 times (Lin et al., 2017). Analysis of the TCGA-LIHC dataset revealed that HMGCR is upregulated in HCC patients compared to healthy individuals. Furthermore, the expression of HMGCR in liver tissues of clinical HCC patients is significantly higher and closely associated with the progression of HCC (Lin et al., 2017).

The regulation of HMGCR is interconnected with cellular signaling pathways, and overactivation of HMGCR may promote the activation of these signaling pathways, thereby participating in biological processes such as proliferation, anti-apoptosis, invasion, and metastasis of liver cancer cells (Sadowska et al., 2023). As demonstrated in previous studies, the PDZ-binding motif could effectively promote HCC growth through the TEA domain transcription factor 2-ANLN/kinesin family member 23 pathway. The expression of the PDZ-binding motif in HCC is regulated by HMGCR (Saito et al., 2023). Existing studies have found that inhibiting HMGCR can suppress myelocytomatosis oncogene (MYC) phosphorylation through Rac GTPase, thereby inhibiting tumor initiation and *in vivo* growth in MYC-induced HCC transgenic models and human-derived HCC cell lines (Cao et al., 2011). Heat shock protein 90 (HSP90) has been reported to promote HCC cell growth and inhibit apoptosis. The mechanism is that HSP90 can interact with HMGCR to regulate its protein expression levels by inhibiting protein degradation, and ultimately promote the progression of HCC (Dong et al., 2019). The role of the forkhead box protein M1 (FoxM1) transcription factor in the development of HCC has been well documented. The knockdown of HMGCR can reduce the expression of FoxM1, suggesting that HMGCR can inhibit the development of HCC by regulating the expression of FoxM1 (Ogura et al., 2018).

Statins are a class of classical HMGCR inhibitors. A case report has shown significant clinical efficacy in reducing the incidence and mortality of cardiovascular diseases in various high-risk populations, making them the main drugs for treating hypercholesterolemia (Polmann et al., 2023). Statins can prevent the occurrence and recurrence of HCC by treating a risk factor hypercholesterolemia (Tran et al., 2020; Kim et al., 2018; Tokushige et al., 2011). Simvastatin, an inhibitor of the mevalonate pathway, effectively abolishes tumor growth in nude mice, and clinical data indicate that patients with lower levels of the mevalonate pathway enzyme HMGCR have better prognoses (Yi et al., 2020). Using various statins, including fluvastatin, pravastatin, simvastatin, atorvastatin, and rosuvastatin, histological lesions were analyzed through a grading and staging system for MASH. The results showed that statin treatment could prevent the development of MASH, and the effect of each statin on improving MASH was independent of its cholesterol-lowering effects. Statins inhibit the occurrence of HCC by regulating Yes-associated protein (YAP) through Rho GTPases (Yi et al., 2020). However, statins do not reduce the incidence of MAFLD-associated HCC in patients (Yi et al., 2020). For example, atorvastatin failed to reverse N-nitroso diethylamine-induced HCC in mice (Braeuning et al., 2014). Currently, the potential role of cholesterol-lowering drugs in the treatment of HCC is a topic of active discussion. It has been shown that miR-206 can disrupt the positive feedback loop between cholesterol synthesis and the pentose phosphate pathway by simultaneously targeting *HMGCR* and *G6PD*, thereby reducing cholesterol and DNA synthesis required for malignant hepatocyte growth and proliferation (Hu et al., 2023). This suggests that the impact of drugs on various pathways within the organism may be a key factor affecting efficacy.

Although cholesterol-related protein inhibitors demonstrate potential in regulating metabolic homeostasis, their clinical application is confronted with multiple paradoxes. Statins, by inhibiting HMGCR, significantly reduce LDL-C levels; however, studies have found that up to 82% of young individuals who have experienced myocardial infarction are ineligible for statin therapy (Blankstein and Singh, 2020). The multisociety guidelines on blood cholesterol management issued by the American Heart Association and the American College of Cardiology provide new criteria to achieve more tailored risk assessments, thereby guiding statin prescription in primary prevention (Zeitouni et al., 2020).

### 3.2 SQLE

#### 3.2.1 Structure of SQLE

SQLE is an enzyme protein composed of 574 amino acid residues, found in various tissues including the liver, kidney, muscle, and skin. The molecular interactions of the SQLE enzyme-substrate complex must be very precise to tightly control the regional and stereochemistry of the epoxidation reaction (Abe et al., 2007). The oxygen atom generated in this process becomes the characteristic hydroxyl group of cholesterol. The reaction requires molecular oxygen, flavin adenosine dinucleotide (FAD), nicotinamide adenine dinucleotide phosphate (NADPH), and electron transfer partners, including NADPH-cytochrome P450 reductase (Porter, 2015). Delivery of the substrate to the active site of SQLE requires anionic phospholipids and lipid transfer proteins, namely, apolipoprotein factor (Chin and Bloch, 1984).

Its N-terminal truncated protein contains three distinct structural domains, namely, the FAD-binding domain, substrate-binding domain, and C-terminal helix membrane-binding domain. The hydrophobic N-terminal structural domain is essential when purifying the full-length form of mammalian SQLE (Padyana et al., 2019). The crystallographic structure of the hydrophobic N-terminal structural domain lacks the first 117 amino acids, which includes the cholesterol regulatory domain. Cholesterol accelerates the degradation of SQLE through this N-terminal region of 100 amino acids (Chua et al., 2017).

#### 3.2.2 Regulatory factors of the SQLE level

The activity and expression of SQLE are regulated by multiple factors. Transcription factors such as SREBP, LXR, pregnane X receptor (Gwag et al., 2019), and hepatocyte nuclear factor (HNF) 4α bind to the promoter region of the SQLE gene and regulate the transcriptional activity of SQLE (Kim et al., 2002; Repa et al., 2002; Thomas et al., 2013). SQLE activity can be negatively feedback-regulated by its product, lanosterol. When lanosterol concentration is high, it will inhibit the activity of SQLE to maintain the balance of cholesterol synthesis; Additionally, the activity of SQLE is also regulated by the concentration of its substrate, squalene. Higher concentrations of squalene can promote SQLE activity, thereby increasing cholesterol synthesis (Llorentecortes et al., 2007).

#### 3.2.3 SQLE as a therapeutic target in liver injury

Research has found that SQLE is the most highly expressed metabolic gene in MAFLD-HCC patients. Liver-specific *Sqle* knockout in mice inhibited tumor growth, increased cytotoxic CD8^+^ T cells, suggesting that SQLE promotes immune suppression in MASH-HCC (Llorentecortes et al., 2007). Increased SQLE levels can elevate the NADP^+^/NADPH ratio, leading to a series of events: firstly, oxidative stress-induced expression of DNA methyltransferase 3 alpha (DNMT3A) increases, which then mediates the epigenetic silencing of phosphatase and tensin homolog and activation of the mammalian target of rapamycin, ultimately resulting in the occurrence and development of HCC (Liu et al., 2018). It has also been found that SQLE can promote bovine serine-threonine kinase receptor-associated protein transcription and transforming growth factor-β/small mother against decapentaplegic (TGF-β/SMAD) signaling or positively regulate extracellular regulatory protein kinase signaling to drive HCC development and progression (Zhang et al., 2023b). Alternatively, SQLE directly binds to carbohydrase 3, inducing activation of SREBP1C, acetyl-CoA carboxylase, FASN, and SCD1 expression, and driving the initiation and progression of MASH through *de novo* lipogenesis (Sui et al., 2015).

Terbinafine, an FDA-approved antifungal drug, significantly inhibited SQLE-induced growth of HCC cells and attenuated tumor development in xenograft models and *Sqle* transgenic mice (Liu et al., 2018). Another study showed that pharmacological inhibition of SQLE by terbinafine limited liver tumorigenesis in MAFLD-induced liver tumors in *Trp53* knockout mice (Sun et al., 2021). In an advantageous position, the lipophilic portion of terbinafine is located vertically within the SQLE binding pocket, with the tert-butyl group toward its center. Such a position results in a change in SQLE conformation and prevents the natural substrate from being able to bind to the active site of the enzyme (Liu et al., 2018). Moreover, the binding energy of terbinafine targeting SQLE is higher than −3.73 kcal/mol (Lee et al., 2021). Although SQLE inhibitors such as terbinafine can block the oxidation of squalene, clinical trials have identified an associated risk of elevated liver enzymes. The pathophysiology of terbinafine-induced hepatotoxicity remains unclear. Bile duct injury or other biliary secretion disorders, which impede bile flow, appear to be a primary factor in liver damage induced by terbinafine (Teixeira et al., 2024).

NB-598 competitively inhibits SQLE in cultured cells and induces an increase in LDLR mRNA and protein (Hidaka et al., 1991). Moreover, NB-598 has been reported to reduce hepatic secretion of triacylglycerol-rich lipoproteins and inhibit hepatic cholesterol synthesis by enhancing intracellular degradation of apolipoprotein B, thereby exerting a hypolipidemic effect (Masahiro et al., 1993).

HMGCR and SQLE are key enzymes in the cholesterol biosynthetic pathway, regulating cholesterol synthesis and metabolism. Their aberrant expression or activity in liver diseases may lead to dysregulated cholesterol metabolism, impacting liver health, promoting lipid accumulation, and hepatocellular damage. Therefore, investigating the roles of these enzymes in liver diseases can provide insights into the relationship between dysregulated cholesterol metabolism and liver disorders, and may offer valuable clues for the development of novel therapeutic strategies.

### 3.3 SREBPs

#### 3.3.1 Structure of SREBPs

SREBPs have three structural domains. The N-terminal cytoplasmic structural domain contains a basic helix-loop-helix DNA-binding motif, which can function as a transcription factor when dissociated from SREBPs into the nucleus. The central membrane-anchored structural domain contains two transmembrane α-helices and the C-terminal cytoplasmic regulatory structural domain (Goldstein et al., 2002). SREBP cleavage activating protein (SCAP) has eight transmembrane α-helices and a large C-terminal cytoplasmic structural domain that binds to the C-terminal cytoplasmic regulatory domain of SREBPs (Yan et al., 2021). The SCAP-SREBPs complex is translocated from the ER to the Golgi complex via vesicles. At the Golgi, SREBPs undergo two proteolytic trimmings and the N-terminus enters the nucleus, termed nuclear SREBPs (nSREBPs). The nSREBPs that enter the nucleus transcriptionally regulate the expression of genes that contain sterol regulatory elements in the promoter region, such as HMGCR.

#### 3.3.2 Regulatory factors of SREBPs level

Several transcription factors can directly or indirectly affect the transcriptional activity of SREBPs. For example, proteins such as SCAP and SREBP signal pathway-regulated gene 1 are closely related to the regulation of SREBPs activity (Foufelle and Ferré, 2002). The activity of SREBPs is also regulated by protein ubiquitination and degradation. X-box binding protein 1 (XBP1), initially characterized as a basic leucine zipper transcription factor, exists in unspliced (XBP-u) and spliced (XBP1-s) forms. Existing studies have found that XBP1-u co-localizes with SREBP2 and can inhibit its ubiquitination and proteasomal degradation (Wei et al., 2022). In addition, in hepatocytes exposed to high glucose, AMPK can stimulate phosphorylation at Ser372, inhibiting the cleavage and nuclear translocation of SREBP1 and suppressing the expression of SREBP1 target genes (Li et al., 2011). The mTORC1 inhibitor rapamycin blocks nuclear accumulation of the mature form of SREBP1 and expression of SREBP target genes (Porstmann et al., 2008). miR-122 and miR-33 may be key regulators of SREBP1. Silencing or overexpressed miR-122 *in vitro* revealed that the mRNA level and protein level of SREBP1 were correspondingly increased and decreased respectively (Cheung et al., 2008). Whereas, lack of miR-33 in mice exacerbated high-fat diet-induced obesity and hepatic steatosis and significantly upregulated SREBP1 expression, suggesting that SREBP1 is a target of miR-122 and miR-33 (Horie et al., 2013). Additionally, studies indicate that overexpression of angiotensinogen in the renin-angiotensin system can also inhibit the expression of SREBP1 and its downstream molecules (Tao et al., 2019).

#### 3.3.3 SREBPs as therapeutic targets in liver injury

Cross-analysis of multiple databases and clinical observations confirm that SREBP levels are elevated in HCC, and the overexpression of SREBF2 eliminates the tumor-suppressive activity of lecithin-cholesterol acyltransferase (LCAT) (Tao et al., 2019). The epithelial-mesenchymal transition (EMT) plays a crucial role in the heterogeneity and metastasis of tumors. Studies have reported that SREBP2 can promote the invasion and metastasis of HCC cells by inducing EMT (Zhang et al., 2023a).

Fatostatin (125B11) is a specific inhibitor of SREBP activation (Ma et al., 2024), which can inhibit the translocation of SREBPs from the ER to the Golgi apparatus by binding to SCAP, thereby inhibiting the activation of SREBP1 and SREBP2. Previous studies have shown that fatostatin reduces obesity, improves fatty liver, and lowers hyperglycemia in obese mice by decreasing the transcription of lipogenic genes and reducing triglyceride storage (Kamisuki et al., 2009).

Besides, recent studies have found that many herbs or food extracts such as saffron (Luo et al., 2019), bovine mushroom extract (Peng et al., 2017), and green tea polyphenols (Tan et al., 2017) can also reduce hepatic steatosis by phosphorylating activated AMPK and inhibiting SREBP1 expression.

Overall, SREBPs regulate the balance between cholesterol synthesis and uptake by feedback control of cellular cholesterol levels, maintaining cholesterol homeostasis. Dysregulation of SREBP activity may lead to abnormal cholesterol metabolism, as well as various metabolic disorders such as diabetes and obesity.

### 3.4 NPC1L1

#### 3.4.1 Structure of NPC1L1

NPC1L1 is a transmembrane protein composed of 1,332 amino acids, featuring 13 transmembrane domains and multiple glycosylation sites, including an NPC domain and a sterol sensing domain (Hu et al., 2021; Poongavanam et al., 2019). NPC1L1 passes through the extracellular N-terminal structural domain, causing cholesterol to aggregate and bind to the sterol-sensing structural domain of NPC1L1. The above complexes undergo endocytosis and interact with clathrin complex 2 to form endosomes, subsequently transported to late endosomes and lysosomes for digestion (Huang et al., 2020). Most of the remaining NPC1L1 is transported to the endocytosis recycling compartment where it is detached from cholesterol and returned to the cell surface to restart cholesterol transport (Huang et al., 2020).

#### 3.4.2 Regulatory factors of NPC1L1 level

Transcription factors closely associated with cholesterol metabolism such as SREBP2, peroxisome proliferators activated receptor (PPAR) α and δ (Rezaei et al., 2022), and HNF1α (Yang et al., 2023), have been reported to regulate the expression of NPC1L1. These factors have been shown to directly bind to the NPC1L1 promoter and synergistically increase the activity of the NPC1L1 promoter.

#### 3.4.3 NPC1L1 as a therapeutic target in liver injury

A comprehensive bioinformatics analysis using the TCGA databases revealed that both NPC1L1 expression and the frequency of genetic variations were significantly elevated in HCC compared to the control group. Furthermore, NPC1L1 demonstrated correlations with pathological stages, differentiation grades, and various clinical parameters (Barretto et al., 2014). Hepatitis C virus (HCV) can be transmitted to naive cells via free viral particles and cell-to-cell transmission, while intercellular spread of HCV and exacerbation of HCV depend on NPC1L1 (Barretto et al., 2014). Furthermore, studies have shown that heating cholesterol-rich oxysterols can exacerbate high-fat diet-induced steatosis in a liver NPC1L1-dependent manner (Yamanashi et al., 2022).

Existing studies have shown that the cholesterol uptake inhibitor ezetimibe can effectively inhibit the proliferation of HCC cells by targeting NPC1L1 to inhibit the PI3K/AKT/mTOR signaling pathway to induce the occurrence of non-apoptotic programmed cell death (Yin et al., 2023). Ezetimibe is effective in lowering blood lipids, but there is a risk of adverse reactions such as unstable angina, rhabdomyolysis and autoscopy (Han et al., 2024).

### 3.5 Aster

#### 3.5.1 Structure of aster

Lipid transfer proteins anchored at membrane contact sites (Lam) family include three members in mammals (GRAMD1a/Aster-A, GRAMD1b/Aster-B, and GRAMD1c/Aster-C). They share an N-terminal GRAM and a StART-like domain, followed by a C-terminal transmembrane domain. Through interactions between their transmembrane domain and amphipathic helices within the lumen, Aster proteins form homodimeric and heterodimeric complexes. When localized at the ER membrane contact sites throughout steady-state conditions, they swiftly transport cholesterol and anionic lipids proximal to the plasma membrane (PM) to the ER membrane contact sites, mediated by the GRAM domain-dependent recognition (Sandhu et al., 2018).

#### 3.5.2 Regulatory factors of aster level

Earlier work showed that the GRAM domains of the Aster proteins are recruited to the PMs of living cells in response to cholesterol loading and facilitate the formation of PM-ER contacts, where sterol transfer occurs (Sandhu et al., 2018). Increased cholesterol levels are sensed by changes in phosphatidylserine (PS) presentation. In the plasma membrane, the anionic lipid PS can contribute to the generation of a robust Aster-GRAM structural domain binding by incorporation into liposomes. Additionally, existing studies have found that Gramd1b is transcriptionally regulated by LXR in macrophages (Ferrari et al., 2020).

#### 3.5.3 Aster as a therapeutic target in liver injury

Existing research on GRAMD1a/Aster-A, GRAMD1b/Aster-B, and GRAMD1c/Aster-C is limited. However, their roles in the liver, particularly in MAFLD, have attracted scholarly attention (Ferrari et al., 2020). Interestingly, analysis using the GEPIA 2.0 database (http://gepia2.cancer-pku.cn/#index) revealed a significant increase in GRAMD1a/Aster-A expression in HCC tissues compared to normal liver tissues, while no such increase was observed for GRAMD1b/Aster-B and GRAMD1c/Aster-C.

Aster-B/C activates soluble adenylyl cyclase, triggering a calcium RhoA-mediated pathway that suppresses β-TrCP/proteasome-mediated TAZ degradation. In mice fed with a cholesterol-rich MASH-inducing diet, hepatocyte-specific silencing of Aster-B/C, sAC, or RhoA decreased TAZ and ameliorated fibrotic MASH (Wang et al., 2020).

The inhibitor AI-3d, which had been shown previously to inhibit Aster-mediated non-vesicular transport *in vitro*, reduced cholesterol transport to the ER and expanded the pool of accessible cholesterol at the plasma membrane of intestinal enteroids. In this study, Simvastatin also demonstrated a similar effect. Finally, treatment of mice with AI-3d inhibited cholesterol absorption (Ferrari et al., 2023; Xiao et al., 2021).

NPC1 and NPC2 are key proteins in the cholesterol transport pathway, and mutations in them can lead to lipid storage disorders. OSBP interacts with Aster protein and plays important roles in cholesterol transport and membrane organization, regulating cholesterol homeostasis. A deeper understanding of the interactions between NPC1/2, OSBP, and Aster in the cholesterol transport pathway can help uncover the pathogenic mechanisms of liver diseases, such as MAFLD.

### 3.6 OSBP/ORPs

#### 3.6.1 Structure of OSBP/ORPs

Proteins with high homology to the structure of OSBP are called OSBP-related proteins (ORPs). OSBP and ORPs form a family of proteins - the ORPS family. The ORPS family of protein sequences has some common features. One of them is that they all have a structural domain OSBP-related ligand-binding domain (ORD) at the C-terminus of about 150–200 amino acids in size with high similarity (Olkkonen and Ikonen, 2022). There is a highly conserved sequence in ORD, which is called fingerprint sequence of ORPs, and it is highly conserved among all ORPs protein sequences. It is highly conserved among all ORPs protein sequences. There is also a conserved FFAT motif in the ORD domain, which mediates the interaction of ORPs family proteins with vesicle associated membrane protein (VAMP)-associated proteins on the ER, which is important for ORPs localization at the plasma membrane (Perry and Ridgway, 2006).

#### 3.6.2 Regulatory factors of OSBP/ORPs level

The function of OSBP in tethering organelles and transporting lipids is coupled through a four-step cycle. The membrane tethering by the PH domain interacting with Golgi phosphoinositide PI(4)P, and the interaction of the FFAT motif with the ER protein VAP-A, facilitate sterol transfer by the lipid transfer domain (ORD), followed by the reverse transfer of PI(4)P by the ORD. Subsequently, PI(4)P is hydrolyzed by the ER protein Sac1. The energy derived from PI(4)P hydrolysis drives sterol transfer and provides negative feedback in the case of PI(4)P depletion (Mesmin et al., 2013).

#### 3.6.3 OSBP/ORPs as a therapeutic target in liver injury

For the OSBP/ORP family, different subtypes exhibit varying expression levels in patients with liver injury. Dysregulation of several ORP family members is implicated in cancers, ORP3, -4, -5 and -8 being thus far the most studied examples (Wang et al., 2014). CHO-OSBP cells showed a 40%–60% decrease in acyl-CoA: cholesterol acyltransferase activity and mRNA, a 50% elevation in mRNA for three sterol-regulated genes [LDL receptor, 3-hydroxy-3-methylgluraryl (HMG)-CoA reductase and HMG-CoA synthase], and an 80% increase in [^14^C]acetate incorporation into cholesterol, leading to the occurrence of fatty liver (Lagace et al., 1997; Lin et al., 2023; Zhou et al., 2011). Furthermore, OSBP plays a critical role in HCV replication and membranous web integrity. OSBP is recruited to membranous webs in a PI 4-kinase-dependent manner, and both factors have been found to regulate cholesterol trafficking to the web (Wang et al., 2014).

Schweinfuran glycosides (SWs) are naturally occurring prenylstilbenes with favorable anticancer properties (Wright et al., 2021). Existing studies have found that they control the intracellular distribution of cholesterol through OSBP. OSBP-IN-1 is a SW derivative. In cells, the interaction between OSBP-IN-1 and OSBP reduced membrane cholesterol level and delayed post-Golgi trafficking (Péresse et al., 2020). It has been reported that delayed post-Golgi trafficking induces ER stress, suppresses both PI3K activation and mTOR/RheB complex formation, and subsequently leads to the inhibition of PI3K/AKT/mTOR signaling. These signaling events can lead to apoptosis (Bao et al., 2015).

Another study found that itraconazole can inhibit enterovirus and HCV replication by targeting OSBP and OSBP-related protein 4 (ORP4) (Jeroen et al., 2015).

### 3.7 NPC1/2

#### 3.7.1 Structure of NPC1/2

The Niemann-Pick disease type C1 (NPC1) protein consists of 1,278 amino acids and is mainly localized on the endosomal and lysosomal membranes. Its structure is complex, including the N-terminal domain, domain C (also known as middle lumenal domain), domain I (also known as carboxy-terminal domain), and 13 transmembrane domains. Among them, TM2-6 is considered the sterol-sensing domain (SSD), while TM8-12 is classified as the SSD-like domain. Previous studies have shown that SSD can bind to cholesterol molecules, which is crucial for the function of NPC1 in cholesterol metabolism (Qian et al., 2020a).

Mature human NPC2 protein contains 132 amino acid residues with a theoretical molecular weight of 14.5 kDa. NPC2 protein is localized in lysosomes and is a single-domain luminal protein. Despite its relatively simple structure, NPC2 also plays an important role in cholesterol metabolism, especially in the transport and metabolism of cholesterol in lysosomes (Vanier and Millat, 2004). Cholesterol-rich low-density lipoprotein (LDL) is first internalized into cells through LDLR on the cell membrane. In endosomes or lysosomes, NPC2 protein extracts cholesterol from LDL and then delivers it to the membrane-bound NPC1 protein. Finally, NPC1 interacts with downstream proteins to further transport cholesterol to other parts of the cell, thereby exerting biological functions (Gong et al., 2016).

#### 3.7.2 Regulatory factors of NPC1/2 level

One of the most abundant membrane proteins in lysosomes, lysosome-associated membrane protein 2 (LAMP2), is believed to receive cholesterol from NPC2, thereby assisting in the efflux of cholesterol from lysosomes driven by NPC1 (Heybrock et al., 2019). LAMP protein can serve as a reservoir for the cholesterol extracted by NPC2 from the inner membrane of lysosomes before NPC1 exports cholesterol from lysosomes. It has been demonstrated that LAMP2 tightly binds to the N-terminal domain of NPC1 and also binds cholesterol in the same direction as this domain (Li and Pfeffer, 2016). NPC2 functions by binding cholesterol in the lysosomal lumen and delivering it to other proteins, such as membrane-bound NPC1, to facilitate transportation across the lysosomal membrane. Studies have shown that NPC2 promotes the delivery and removal of cholesterol in NPC1, as well as the transport of cholesterol between membranes (Schneede et al., 2011). Other studies have found that bis (monoacylglycerol) phosphate (BMP) and sphingomyelin (SM) are present in lysosomes and late endosomes. When bound to membranes, NPC2 collides with membranes, BMP regulates its cholesterol transport efficiency, and SM strongly inhibits cholesterol translocation by NPC2 (Schneede et al., 2011).

#### 3.7.3 NPC1/2 as a therapeutic target in liver injury

NPC1 disease is an autosomal-recessive cholesterol storage disorder. Recent studies have shown that NPC1 mRNA is highly expressed in HCC tissues, as evidenced by data from the TIMER, UALCAN, Oncomine, and HCCDB databases. Furthermore, clinical HCC tissues demonstrate that NPC1 expression in tumor tissues is higher than in adjacent tissues, suggesting that NPC1 may serve as a novel pan-cancer biomarker (Ebner et al., 2018). In addition to other symptoms, NPC1 patients develop liver dysfunction and hepatosplenomegaly. The livers of Npc1^−/−^ mice showed hepatic cholesterol sequestration resulting in consecutive liver injury, an increase in the expression of lipogenic genes such as HMG-CoA, a decrease in the expression of lipolytic genes such as PPARα and acyl-CoA oxidase 1, and a decrease in the expression of lipid transporter genes such as ACAT1, ABCA1, and fatty acid transport protein 2 (Ebner et al., 2018).

NPC1 is a critical intracellular receptor for viral infection. The cellular entry of quasi-enveloped variants of hepatitis A virus and hepatitis E virus is also linked to NPC1 (Ahmad et al., 2023). VTS-270 has been shown to alleviate cholesterol and glycosphingolipid accumulation caused by NPC1 mutations in Phase 1/2 clinical trials (Ebner et al., 2018). Pitavastatin also regulates cholesterol transport by inhibiting NPC1 through the PI3K/AKT pathway (Ebner et al., 2018). In the MASH model, NPC2 is ineffective in modifying robust liver MASH endpoints. Nevertheless, data suggest that hepatic ABCA1 expression and reverse cholesterol transport are upregulated by NPC2 treatment, presenting a putative therapeutic effect in diseases associated with lipid metabolism disorders (Christensen et al., 2018).

### 3.8 ACAT

#### 3.8.1 Structure of ACAT

ACAT is an integral membrane protein localized in the ER. In mammals, two ACAT genes have been identified, ACAT1 and ACAT2. ACAT1 is responsible for the formation of cholesteryl esters in the brain, adrenal glands and kidneys, while ACAT2 is predominantly expressed in the liver and intestines.

ACAT1 is a tetramer composed of two homodimers. Each monomer contains nine transmembrane helices, with six forming a cavity that accommodates endogenous acyl-CoA. The cavity also contains a histidine, which is required for its catalytic activity (Guo et al., 2005). ACAT1 senses free cholesterol through its metastable site. At low cholesterol concentrations, ACAT1 does not efficiently catalyze esterification. However, at high concentrations, excess cholesterol will denature to promote esterification. This mechanism would ensure that ACAT1 activity can be regulated by the concentration of free cholesterol in the membrane to maintain cholesterol homeostasis in the ER (Long et al., 2020).

The membrane topology of ACAT2 is similar to that of ACAT1. Its entrance site is occupied by a cholesterol molecule, and another site is used for the allosteric activation of ACAT2 (Long et al., 2021).

#### 3.8.2 Regulatory factors of ACAT level

A variety of factors such as hormones, cytokines, and nutritional factors, among other signaling molecules, can regulate the expression and enzymatic activity of ACAT, altering the balance of cholesterol metabolism. For example, tumor necrosis factor alpha has been shown to upregulate ACAT expression and increase the process of cholesterol esterification (Lei et al., 2009). Estrogen has also been reported to regulate ACAT protein levels (Kavanagh et al., 2009). Whereas HNF1 was shown to be an important liver-specific cis-acting element of the human ACAT2 gene. Transcription factors HNF1α and HNF1β can regulate its expression in the liver by binding to the ACAT2 promoter (Pramfalk et al., 2005).

Cellular signaling kinases, AMPK, and cytochrome P450 enzymes have been shown to directly or indirectly regulate ACAT through different pathways (Hanke et al., 2008). For example, phosphoglycerate mutase 5, as a mitochondrial serine/threonine phosphatase, mediates the dephosphorylation of isocitrate dehydrogenase 1 at S336, which induces the acetylation of isocitrate dehydrogenase 1 mediated by ACAT1 at K337 (Zhu et al., 2020).

Additionally, current research has found that miR-21 has a regulatory effect on the 3′-untranslated region of rat ACAT1 (Chanyshev et al., 2020). In the background of apolipoprotein E deficiency, the deletion of the vasopressin II receptor gene can also promote the downregulation of ACAT1 (Bousette et al., 2009).

#### 3.8.3 ACAT as a therapeutic target in liver injury

In the normal adult liver, ACAT2 expression is very low, but it is significantly elevated in liver samples from HCC patients, suggesting that the increased expression of ACAT2 may serve as a novel biomarker for HCC (Bousette et al., 2009). HCC is associated with the dysregulation of glycerophosphate acyltransferase (GNPAT). Available studies found that ACAT1 acetylates GNPAT at K128, thereby inhibiting the ubiquitination and degradation of GNPAT mediated by the E3 ubiquitin ligase *TRIM21*. Knockdown of ACAT1 and loss of GNPAT acetylation, as mediated by shRNA, inhibit xenograft and carbon tetrachloride-induced HCC lipid metabolism and tumor progression. ACAT1-mediated GNPAT acetylation plays a crucial role in hepatocarcinogenesis (Gu et al., 2020). In addition, the lack of ACAT1 deficiency significantly increased the levels of free cholesterol in hematopoietic stem cells, enhanced Toll-like receptor protein 4, and downregulated the expression of TGF-β pseudo-receptor bone morphogenetic proteins and activin membrane-bound inhibitory factor, thus activating the sensitivity of hematopoietic stem cells to TGF-β, exacerbating liver fibrosis (Tomita et al., 2014b).

Pharmacological and gene regulatory strategies targeting ACAT have now been extensively investigated. ACAT inhibitors, such as avasimibe, reduce fat deposition and improve hepatic steatosis in the livers of mice in a high-fat animal model (Jiang et al., 2019; Zhu et al., 2021). Further research may reveal the potential of ACAT inhibition in the treatment of liver injury (Shibuya et al., 2015).

Accumulation of cholesterol after HCV infection stimulates the production of cholesteryl esters, the main component of lipoviral particles. Existing study has found that the potent ACAT inhibitor TMP-153 can reduce HCV particle density and infectivity by inhibiting cholesterol ester synthesis, thereby reducing the risk of sexual transmission (Read et al., 2014). Furthermore, recent food science studies have found that extracts of many herbs or foods such as piperine (Matsuda et al., 2008) may also reduce the accumulation of lipid droplets in the liver by inhibiting ACAT enzyme activity.

Cholesterol esterification is a critical metabolic process in which the enzyme ACAT plays a pivotal role. The activity of ACAT is regulated by various factors, including tumor necrosis factor α, estrogen, HNF1, extracellular signal-regulated kinases, AMPK, cytochrome P450 enzymes, and miR-21, among others. The activity of ACAT in the liver is closely associated with liver damage. Studies indicate that abnormal activity of ACAT may exacerbate the development of liver diseases such as fatty liver, MAFLD, and others.

### 3.9 ABCA1

#### 3.9.1 Structure of ABCA1

The ATP-binding cassette (ABC) transporter protein family is one of the largest membrane protein families found in a wide range of organisms from prokaryotes to humans. ABC transporter proteins are also known as efflux pumps because they mediate the ATP hydrolysis-driven transmembrane transport of various internal and external biomolecules (Wang et al., 2021). ABC transporters feature a characteristic structure composed of at least four domains: two ABC domains (or nucleotide-binding domains) with highly conserved sequence motifs and two transmembrane domains. Additional domains can fuse with these core elements to confer regulatory functions. The structural similarity of these domain structures supports the coordination of a series of nucleotide and substrate-dependent conformational changes in ABC transporters, whether they are importers or exporters, leading to substrate translocation across the membrane through an alternating access model (Chen et al., 2022).

It is worth noting that ABCA1-mediated phospholipid efflux transport has been proposed to be not an alternate entry model, but a lateral entry model of transport. This lateral entry model allows substrates to enter the substrate-binding pocket of the transmembrane region from the inner leaflet of the cell membrane even when the transmembrane region is outwardly open (Qian et al., 2017).

#### 3.9.2 Regulatory factors of ABCA1 level

Current research indicates that transcription factors and cytokines can regulate the expression of ABCA1. There are two main types of ABCA1 mRNA: one found in the intestines, containing a 222 bp exon designated as the P-type. The other is the L-type of the transcript in the liver, which lacks the aforementioned exon (Tamehiro et al., 2007). Sterols can increase P-type promoter activity (Wong et al., 2006), while inhibition of endogenous oxysterol LXR ligand formation or increased binding of SREBP2 to the E-box element can reduce P-type promoter activity (Zeng et al., 2004). The L-type promoter is activated by binding to SREBP2. Both estrogen receptor α and PPARγ coactivator 1α can positively regulate ABCA1 translation by binding to transcripts (Bao et al., 2023; Chen et al., 2023a).

In addition, maternal uptake of a high-calorie diet can inhibit the expression of ABCA1 by decreasing the methylation level of ABCA1 in the offspring (Christensen et al., 2018). miR-30a-3p can inhibit the expression of ABCA1 by directly targeting the 3′ untranslated region of ABCA1 (Chen et al., 2023b).

#### 3.9.3 ABCA1 as a therapeutic target in liver injury

It has been found that the cholesterol transporter ABCA1 is upregulated in tumor monocytes/macrophages, leading to the generation of immature and immunosuppressive monocytes/macrophages. Large amounts of ABCA1 and monocytes/macrophages in HCC reduce CD8^+^ T cell infiltration, which contributes to the poor prognosis of HCC (Li et al., 2023).

Researchers synthesized CircRNA_0001805 targeting miR-106a-5p/miR-320a as an upstream inhibitor of ABCA1 to regulate MAFLD progression (Li et al., 2021). The ABCA1 inhibitor glibenclamide has been shown to ameliorate high-fat diet-induced non-alcoholic fatty liver disease in rats by lowering levels of blood glucose, triglycerides, cholesterol, DNA damage, apoptosis, and inflammation markers (Dwivedi and Jena, 2020). Another study showed that glibenclamide can promote ROS-dependent activation of the c-Jun N-terminal kinase pathway, inducing apoptosis in HCC cells (Yan et al., 2017).

Probucol, another ABCA1 inhibitor, is a novel antihyperlipidemic drug with potent antioxidant activity that prevents lipid oxidation. A small observational study that included eight patients with MAFLD showed a significant reduction in aminotransferase levels and improvement in liver histology with Probucol (Merat et al., 2008). A randomized controlled trial also demonstrated that probucol effectively reduces alanine aminotransferase levels in patients with MASH (Merat et al., 2003). However, due to its potential to lower high-density lipoprotein levels, the use of probucol in patients with concomitant coronary artery disease needs to be carefully considered (Al-Busafi et al., 2012).

The ABC transporter family is responsible for transmembrane transport of the biomolecules, with a structure comprising ABC domains and transmembrane domains. Factors regulating ABCA1 expression include sterols, estrogen receptor α, and PPARγ agonist 1α. Research indicates that miR-30a-3p can suppress ABCA1 expression by targeting it. Upregulation of ABCA1 in tumor cells affects immune cell function, inhibiting CD8^+^ T cell infiltration and worsening HCC prognosis.

## 4 Discussion

Due to the critical role of cholesterol in cellular physiology and function, alterations in cholesterol homeostasis and metabolism are associated with numerous pathological conditions. In this context, rather than analyzing the role of total cholesterol levels, the focus is on understanding the potential mechanisms of liver injury involving proteins mediated in cholesterol metabolism. Apart from liver injuries directly caused by elevated cholesterol levels, overexpression of most cholesterol metabolism-related proteins (such as HMGCR, SQLE, SREBP2, and ABCA1) may also promote the occurrence of liver injuries through non-cholesterol pathways. Furthermore, this review also discusses the role of drugs targeting proteins involved in cholesterol metabolism in the treatment of liver injuries such as MAFLD, HCV, and HCC.

Most drugs targeting cholesterol metabolism-related proteins have shown promising efficacy in preclinical experiments. The drugs targeting cholesterol metabolism-related proteins highlighted in this paper are presented in Table 1. A cohort study of nearly 1,200 European patients has demonstrated the beneficial effects of statin drugs on hepatic steatosis, steatohepatitis, and fibrosis assessed by liver biopsy (Dongiovanni et al., 2015). The cholesterol uptake inhibitor, ezetimibe, was found to lower plasma cholesterol, attenuate hepatic steatosis in mice fed a high-fat diet, and slow the progression of MAFLD/MASH in animal studies (Muraoka et al., 2011). However, the efficacy benefit of ezetimibe in patients with MAFLD/MASH has conflicting endings. Further comparison reveals that this discrepancy may be due to the lack of multicenter and large-sample clinical trials demonstrating the role of these inhibitors in the treatment of liver injury, and their clinical application remains challenging (Jun and Cheon, 2019). It is worth noting that cholesterol metabolism plays a crucial role in various life activities in the body. In fact, it has been suggested that more appropriate treatment options may be selected for cholesterol metabolism abnormalities occurring at different life stages. For instance, hypercholesterolemia originating from intrauterine development is associated with abnormal epigenetic modifications and the intrauterine programming mechanism of fetal-origin hypercholesterolemia. Interventions targeting pregnant women or early offspring have been proposed to effectively prevent and treat the development of fetal-origin hypercholesterolemia.

When reviewing the role of cholesterol metabolism in liver injury, we primarily identified drugs, miRNAs, transcription factors, and epigenetic factors as upstream factors influencing cholesterol metabolism. However, studies have also suggested that prolonged psychological stress and frequent sleep deprivation among women may lead to hormonal imbalances, consequently elevating levels of high-density lipoprotein cholesterol. Therefore, investigating the impact of external factors such as hormones on cholesterol metabolism-related proteins represents a promising direction for future research.

Furthermore, investigations into the role of these proteins in liver damage have predominantly centered on the characteristics of tumor cells. Tumor cells exhibit distinct features such as unchecked proliferative capacity, genetic and epigenetic variability, self-sufficient growth signaling, resistance to growth inhibition signals, evasion of programmed cell death or senescence, sustained angiogenesis, immune system evasion, tissue invasion, and distant metastasis. Recent research implicates proteins like MYC, HSP90, FoxM1, TGF-β, and EMT in various aspects of tumor cell behavior including uncontrolled proliferation, genetic and epigenetic alterations, autonomous growth signaling, resistance to growth inhibition, immune evasion, tissue invasion, and metastasis.

However, current studies have not yet delved into how resistance to programmed cell death or senescence and sustained angiogenesis could be leveraged to exacerbate liver damage. This gap in knowledge highlights the need for further exploration into the mechanisms underlying liver injury, particularly concerning the interplay between hormonal regulation, protein expression, and the progression of liver pathologies. Expanding research in this area could yield insights into novel therapeutic targets and strategies for managing liver diseases effectively.

In conclusion, cholesterol management therapy seems to be a promising therapeutic approach for liver injuries such as MAFLD and HCC. Therefore, exploring the specific pathogenic mechanisms of liver injuries caused by proteins related to cholesterol metabolism is crucial for providing effective therapeutic targets in clinical settings. However, some mechanisms remain incompletely understood, necessitating further exploration and clinical trial validation.

**TABLE 1:** Involvement of target proteins in diseases and related drugs.

Process	Target	Liver disease	Fold change (disease vs. health)	Drug or compound
Synthesis	HMGCR	MAFLD	2–3	Simvastatin
				Atorvastatin
		Liver fibrosis		Pravastatin
				Rosuvastatin
		HCC		Pitavastatin
				Lovastatin
	SQLE	MAFLD	3–4	Terbinafine NB-598^a^
		MASH		
		HCC		
Uptake	NPC1L1	HCV	1–2	Ezetimibe
		HCC		
Transport	Aster	MAFLD	2–3	Simvastatin AI-3d^a^
		MASH		
	OSBP/ORPs	MAFLD	1–2	Itraconazole OSBP-IN-1^a^
		HCV		
	NPC1/2	MASH	2–3	Pitavastatin VTS-270^a^
		HAV		
		HEV		
Esterification	ACAT	MAFLD	2–4	Avasimibe^a^ TMP-153^a^ Piperine^a^
		Liver fibrosis		
		HCV		
		HCC		
Efflux	ABCA1	MAFLD	1–2	Glibenclamide Probucol
		MASH		
		HCC		
Key protein	SREBPs	MAFLD	1–2	Fatostatin^a^
				Saffron^a^
		HCC		Bovine mushroom extract^a^
				Green tea polyphenols^a^

^a^ Not approved for commercialization by the FDA.

## Glossary

ABCA1: ATP-Binding Cassette Transporter 1
ABC: ATP-Binding Cassette
ACAT1: Acetyl Coenzyme A Acetyltransferase 1
AMPK: Adenosine 5′-Monophosphate-Activated Protein Kinase
BMP: bis (monoacylglycerol) phosphate
DNMT3A: DNA Methyltransferase 3 Alpha
EMT: Epithelial-Mesenchymal Transition
ER: endoplasmic reticulum
FAD: Flavin Adenosine Dinucleotide
FFAs: Free Fatty Acids
FoxM1: Forkhead Box Protein M1
GNPAT: Glycerophosphate Acyltransferase
HCC: Hepatocellular Carcinoma
HCV: Hepatitis C Virus
HDL: High-density Lipoprotein
HMG-CoA: 3-Hydroxy-3-Methyl Glutaryl Coenzyme A
HMGCR: 3-Hydroxy-3-Methyl Glutaryl Coenzyme A Reductase
HNF: Hepatocyte Nuclear Factor
HSP90: Heat shock protein 90
LDLR: Low-Density Lipoprotein Receptor
LXR: Liver X Receptor
MAFLD: Metabolic Dysfunction-associated Fatty Liver Disease
MASLD: Metabolic Dysfunction-associated Steatotic Liver Disease
MVA: Mevalonic Acid
MYC: Myelocytomatosis Oncogene
NADPH: Nicotinamide Adenine Dinucleotide Phosphate
MASH: Metabolic Dysfunction-associated Steatohepatitis
NLRP3: NOD-Like Receptor Family Pyrin Domain Containing 3
NPC1L1: Niemann-Pick C1-like 1
NPC1/2: Niemann-Pick disease type C1/2
ORD: OSBP-related ligand-binding domain
ORP: OSBP-related proteins
OSBP: Oxysterol-binding protein
PM: plasma membrane
PPAR: peroxisome proliferators activated receptor
PS: phosphatidylserine
RIG-I: Retinoic Acid Induced Gene Ⅰ
SCAP: SREBP Cleavage Activating Protein
SM: sphingomyelin
SMAD: Small Mother Against Decapentaplegic
SREBPs: Sterol Regulatory Element Binding Proteins
SQ: Squalene
SQLE: Squalene Epoxidase
SSD: sterol-sensing domain
SW: schweinfuran glycosides
TGF-β: Transforming Growth Factor-β
XBP1: X-box Binding Protein 1
